# Cysteine residues in signal transduction and its relevance in pancreatic beta cells

**DOI:** 10.3389/fendo.2023.1221520

**Published:** 2023-06-29

**Authors:** Blanka Holendova, Lydie Plecita-Hlavata

**Affiliations:** Laboratory of Pancreatic Islet Research, Institute of Physiology, Czech Academy of Sciences, Prague, Czechia

**Keywords:** cysteine, thiol, pancreatic beta cells, posttranslational modifications, redox signaling

## Abstract

Cysteine is one of the least abundant but most conserved amino acid residues in proteins, playing a role in their structure, metal binding, catalysis, and redox chemistry. Thiols present in cysteines can be modified by post-translational modifications like sulfenylation, acylation, or glutathionylation, regulating protein activity and function and serving as signals. Their modification depends on their position in the structure, surrounding amino acids, solvent accessibility, pH, etc. The most studied modifications are the redox modifications by reactive oxygen, nitrogen, and sulfur species, leading to reversible changes that serve as cell signals or irreversible changes indicating oxidative stress and cell damage. Selected antioxidants undergoing reversible oxidative modifications like peroxiredoxin-thioredoxin system are involved in a redox-relay signaling that can propagate to target proteins. Cysteine thiols can also be modified by acyl moieties’ addition (derived from lipid metabolism), resulting in protein functional modification or changes in protein anchoring in the membrane. In this review, we update the current knowledge on cysteine modifications and their consequences in pancreatic β-cells. Because β-cells exhibit well-balanced redox homeostasis, the redox modifications of cysteines here serve primarily for signaling purposes. Similarly, lipid metabolism provides regulatory intermediates that have been shown to be necessary in addition to redox modifications for proper β-cell function and, in particular, for efficient insulin secretion. On the contrary, the excess of reactive oxygen, nitrogen, and sulfur species and the imbalance of lipids under pathological conditions cause irreversible changes and contribute to oxidative stress leading to cell failure and the development of type 2 diabetes.

## 1 Introduction

Cysteine (Cys) exhibits unique character. It contains sulfur in the form of sulfhydryl thiol form which is ionizable, forming a negatively charged thiolate group after deprotonation that further increases its reactivity. This thiol/thiolate group is subject to alkylation by electrophiles and oxidation by reactive oxygen, nitrogen, and sulfur species, resulting in posttranslationally modified forms that may exhibit significantly altered functions (1).

Cys is considered one of the most conserved amino acids in proteins across all species. Strong selection pressure retained Cys residues at functionally important sites and removed them from others (2). The physical and chemical properties of Cys predestine it to be a polar residue (like serine, where the sulfur atom is replaced by oxygen), however, it is considered hydrophobic because it is buried to an increased extent (protection from the solvent), and the number of unpaired Cys residues on the protein surface is minimal (2). Another unique property is its tendency to form clusters with other Cys, as in metal-binding or redox-sensitive proteins, leading to the formation of disulfide bonds. Exposed and isolated Cys are less conserved (1).

## 2 Chemical properties of cysteine

The chemical properties of Cys allow it to be both redox active and strongly nucleophilic due to the large atomic radius of the sulfur atom, the presence of lone pairs of electrons, and the low dissociation energy of the thiol S-H bond.

The thiol groups (R- SH) undergo deprotonation (loss of H^+^), giving the thiolate form R-S^-^. The readiness to provide the proton is given by the pKa, the local pH and electrostatic environment. In proteins, the specific hydrogen bond donors and an electropositive local environment cause a decrease in the pKa value due to the stabilization of the negative thiolate anion, while a hydrophobic environment or an electronegative local environment has the opposite effect (1).

Another important feature is the reductive potential of the thiol group, i.e., the ability to accept or donate electrons. This ability allows the formation of so-called redox pairs, and their interconversion is defined as a reductive or oxidative half-reaction. In biological systems, electron transfer is very dynamic and involves many players. Therefore, many reactions occur even under thermodynamically unfavorable conditions and would never occur in isolation, favoring the kinetic pathways and rates associated with certain reactions (3, 4).

Thiols can produce disulfides in one or more thiol-disulfide exchange processes in intricate biological systems (1). Long assumed to merely serve to stabilize proteins structurally, it is now known that these processes also give rise to many enzymes’ diverse and dynamic functional characteristics (5). The rate-determining stage in the folding process of proteins creating structural disulfide linkages is direct thiol-disulfide exchange. Although *in vivo* enzyme catalysis speeds up the events, spontaneous thiol-disulfide exchange is slow (kinetically inadequate on the folding timescale). Through a sequence of intra- and intermolecular thiol-disulfide exchange processes carried out by oxidoreductases, the folding polypeptide gains a new disulfide bond. Molecular oxygen serves as the oxidizing equivalent in these reactions. A different mechanism for thiol-disulfide exchange involves the oxidative conversion of protein thiols to disulfides and their subsequent reduction. Protein thiols are also significant targets of reactive oxygen species *in vivo*. These processes are crucial for both antioxidant defense and redox regulation of cell signaling, and it is believed that the thiol-disulfide pool is principally responsible for maintaining intracellular redox equilibrium (6). It is now widely acknowledged that thiol-disulfide exchange and thiol oxidation/reduction reactions are dynamic, non-equilibrium processes that are kinetically rather than thermodynamically controlled in cellular systems (7–9). In other words, the partitioning of particular routes depends on relative rates, whereas redox potentials and equilibrium constants just tell whether a reaction is favorable. By fine-tuning the activation energies of reactions that control the outcome of oxidative stimuli or the location of structural disulfides in native proteins, enzymes play a crucial role in these processes.

## 3 Oxidative posttranslational modifications of cysteines

**Figure 1:**
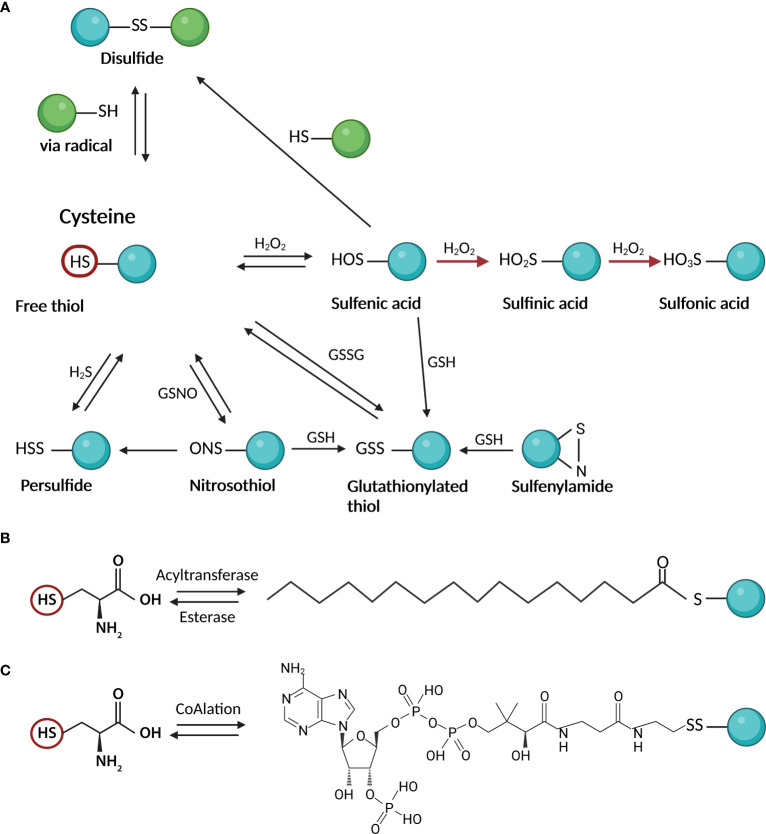
Cysteine posttranslational modifications; **(A)** oxidative modifications: Two-electron oxidation of Cys thiol(ate) groups by hydrogen peroxide, peroxynitrite, and other hydroperoxides, produces sulfenic acid. Sulfenic acid reacts with thiol groups to form disulfides or is further oxidized to irreversible sulfinic or sulfonic acids. Other products of oxidative reactions include S-nitrosothiols and persulfides; **(B)** S-palmitoylation as an example of S – acylation reaction: In the cells, the reaction is mediated by acyltransferases which attach the activated fatty acid-CoA to the Cys moiety. The reversibility of the reaction is ensured by esterases; **(C)** CoAlation: Cysteine thiols of proteins create a reversible mixed disulfide bond called protein CoAlation. This figure was created in Biorender.com (Toronto, Canada).

The availability of different oxidation states of thiol-sulfur allows the formation of a variety of oxidative posttranslational modifications (PTMs) on cysteines, including S-nitrosylation (or S-nitrosation, SNO), sulfhydration (SSH), disulfide bond formation (RS-SR), sulfenylation (SOH), sulfinic acid (SO_2_H), and sulfonic acid (SO_3_H) (10) (**Figure 1A**). Most Cys oxidative PTMs are initiated by reactive oxygen or nitrogen species (ROS/RNS) or sulfane (H_2_S) reacting with the free thiol on a Cys side chain (**Figure 1A**). Two-electron oxidation of thiol(ate) groups, e.g., by H_2_O_2_, peroxynitrite, and other hydroperoxides, produces the simplest oxyacid of sulfur, sulfenic acid. The rate of this reaction can vary from negligibly slow to 10^8^ M^-1^ s^-1^ at active sites of peroxidase (4). Sulfenic acid readily reacts with proximal thiol groups to form disulfides or is further oxidized to irreversible sulfinic or sulfonic acids in the absence of such groups (11). Sulfonic acids can also react with each other to form thiosulfinates or with amine or amide groups to form sulfenylamides (12) (**Figure 1A**). S-nitrosothiols and persulfides are additional oxidative reaction byproducts that are unquestionably significant as signaling intermediates (13, 14). The thiyl radical, another reactive species that can produce a variety of products once generated, can be produced by the one-electron oxidation of thiol groups in the presence of radicals (15).

The reactivity of cysteine to oxidative changes is determined by the proximity of oxidants (in addition to its chemical properties). In β-cells, these are mainly superoxide (O_2_^·−^) and other oxidizing byproducts generated in mitochondria, reduced flavoprotein oxidases or monooxygenases and NADPH oxidase (NOX) family; and NO produced by NOS and combining with O_2_^·−^ also peroxinitrite (16). Endogenous antioxidant system such as superoxide dismutase (SOD), peroxiredoxins (Prdx), glutathione peroxidases (GPX) and compounds such as, glutathione (GSH) and thioredoxin (Trx) are well compartmentalized (17). Together they play a critical role in maintaining redox equilibrium.

## 4 Other posttranslational modifications of cysteines

### 4.1 S-glutathionylation

S-glutathionylation is the attachment of bulky GSH to a cysteine residue via the formation of disulfides, thereby regulating protein functions in response to oxidants (18) (**Figure 1A**). The essential functions of reversible glutathionylation have emerged in physiology, including cardiovascular regulation (18, 19) inflammation and infection (20, 21), apoptosis (22), and cancer (23). S-glutathionylation occurs through nucleophilic sulfur chemistry in which the thiolate anion (S^−^) reacts with oxidized glutathione (GSSG) or reactions of GSH with electrophilic sulfur intermediates, such as sulfenic acid, S-nitrosothiol, or thiyl radical (24). The formation of glutathionylate is also balanced by the activities of various enzymes, including glutaredoxins (Grx) and glutathione transferase pi and omega (GSTP and GSTO) (25).

### 4.2 S-acylation

S-acylation is the covalent attachment of various fatty acids (14-20 carbon atoms) to cysteine residues via a thioester bond (**Figure 1B**). While most lipid modifications to proteins are irreversible, S-acylation is reversible and can be very dynamic (26). Lipid modifications can alter protein transport and membrane localization, interactions, stability, and conformation (26). While S-acylation of cysteines has mainly involved saturated palmitic acid, other studies described Cys modification by oleic acid (27–29), stearic acid (28, 30), and arachidonic acid (29). This can lead to different functional outcomes (29, 30). It depends on the exogenous supply of fatty acids reflecting cellular lipid profiles (31). The added lipid species also may vary from cysteine to cysteine on the same protein (32). The prevalence or functional significance of fatty acid type on S-acylated proteins or modified cysteine in terms of its potential for oxidative modification is not fully understood.

### 4.3 S-CoAlation

In recent years the antioxidant function of a key cellular metabolite, coenzyme A (CoA), has been discovered (33). CoA is an essential cofactor in all organisms and its biosynthesis involves enzymatic conjugation of cysteine, pantothenate and ATP (34). Similar to the antioxidant role of glutathione and glutathionylation of proteins, CoA protects cysteine thiols from hyperoxidation during oxidative stress by forming a mixed disulfide bond, protein CoAlation (33) (**Figure 1C**). Recent research on protein CoAlation has shown that it is a widespread and reversible posttranslational modification. Established cell lines and organisms have been shown to exhibit increased levels of CoAlated proteins upon oxidative or metabolic stress (33). To date, CoAlation was found to modulate the activity of modified proteins and induce significant conformational changes (35).

## 5 Relevance of cysteine oxidation in pancreatic β-cells physiology

For many years, β-cells were attributed as having weak antioxidant defense. However, this was based on comparison with liver or kidney, which are highly specialized detoxifying organs (36). Recently, it was shown that β-cells express Prdxs, Trxs, and SOD1/2. Thus, superoxide formed can be rapidly dismutated to H_2_O_2_, which can then serve as a signaling molecule due to its stability, thiol selectivity, and ability to diffuse through membranes. As mentioned earlier, thiol-containing molecules, Prxs/Trxs exhibit a high affinity for oxidants and are therefore efficient sensors for a prooxidant redox environment. Pancreatic β-cells are glucose sensors responding to high glucose by insulin secretion. Glucose stimulation induces oxidative metabolism, which increases the prooxidant redox status. We and others have shown that short-term glucose stimulation, which increases prooxidant status via cytoplasmic NOX4, is necessary for efficient insulin secretion, whereas long-term stimulation, which tends to induce oxidative stress, can lead to the development of T2D (37, 38). A possible mechanism for the role of prooxidant metabolism in pancreatic β-cells has been proposed (39–46).

The most important, but still technically elusive problem in redox signaling is the compartmentalization of redox status. Cellular localization then determines the source of ROS/RNS/RSS and their proximity to potential cysteine modifications, type of antioxidant defense, and the redox potential of cysteine residues present. Subcellular organelles maintain different pH values and redox potentials as well as concentrations of reactive metabolites that trigger specific redox signals (47, 48). The most important compartments for redox signaling in pancreatic β-cells are the mitochondria, the cytoplasm with the plasma membrane rafts, while oxidation in ER with the Golgi apparatus has more structural functions. Whereas the cytoplasm and mitochondria of quiescent cells have a more reduced environment (-200 and -300mV for the GSH/GSSG redox pair), which gives them a broad probability for redox signaling, e.g. upon induction of proliferation or some other stimulus, ER and Golgi secretion machinery exhibit a more oxidized potential (-150 and -140mV for the GSH/GSSG redox pair) (9, 49). This oxidized status is rather essential for the formation and maintenance of the structural disulfide bonds of the secretory proteins and counteracts redox signaling. It is difficult to determine the reduction potential of individual cellular compartments because different redox pairs are present (GSH/GSSG, Trx, Cys/Cys, etc.). They have individually different midpoint potentials that vary in different organelles. For example, Trx redox pairs are generally more reducing than GSH/GSSG, and while Trx1 shows a redox potential of -280 and -300 mV in the cytosol and nucleus, Trx2 shows an, even more, reducing redox pair of -340 and -360 mV in mitochondria (9, 48). The ability of cysteine to be modified by redox depends on many factors as stated above, but also depends on the presence of molecules with greater reactivity toward thiols such as peroxymonocarbonate. CO_2_ is in equilibrium with bicarbonate, which forms the biological buffer within cells. Bicarbonate can react with peroxide to form peroxymonocarbonate. The rate of its formation increases with decreasing pH (50). Thus, cellular metabolism and its compartmentalization are the main trigger for redox signaling.

### 5.1 Endoplasmic reticulum and Golgi secretion machinery

The importance of the ER/Golgi secretory machinery in β-cells lies in proper insulin maturation and insulin granule formation apart from lipid biosynthesis and folding, glycosylation, trafficking, and secretion of many proteins. Insulin signaling is a key function of β-cells in response to glucose. The acidic environment of ER and the progressive proton gradient from the Golgi to secretory vesicles allow the disulfide bond formation as a PTM not only in insulin molecules. Disulfide formation also mediates biomolecule degradation and ligand dissociation from receptors in the endocytic pathway. Insulin requires 3 disulfide bonds to be formed by the oxidase activity of protein disulfide isomerase (PDI). Electron transfer between PDI and ERO1A provides an oxidizing environment and disulfide bond formation (51). PDI must be reoxidized by Prdx4 in rodents and humans together with Gpx7/8 in humans, with H_2_O_2_ supplied mainly by ERO1 or the present NOX enzymes (52). The tight regulation of ERO1A activity depends on the specific formation of disulfide pairs in the protein backbone (Cys94-Cys99 for the active form vs. Cys94-131 and Cys99-104 for the inactive form) and the quality of folded proteins is thus dependent on the ER redox status (53). Disturbed ER redox homeostasis leads to ER stress, which activates the unfolded protein response. This can multiply ROS production and impair insulin production. Interestingly, Gpx7/8 show low GSH activity due to the absence of domains bound to GSH, but it plays a role in calcium storage (54). Calcium signaling has a key regulatory function for the insulin secretion machinery and functional synchronization of β-cell in the pancreatic islet. The expression of Gpx8 is regulated by Nrf2, which is involved in ER calcium management via the ATPase SERCA (55).

### 5.2 Insulin signaling and redox regulation in β-cells

**Figure 2:**
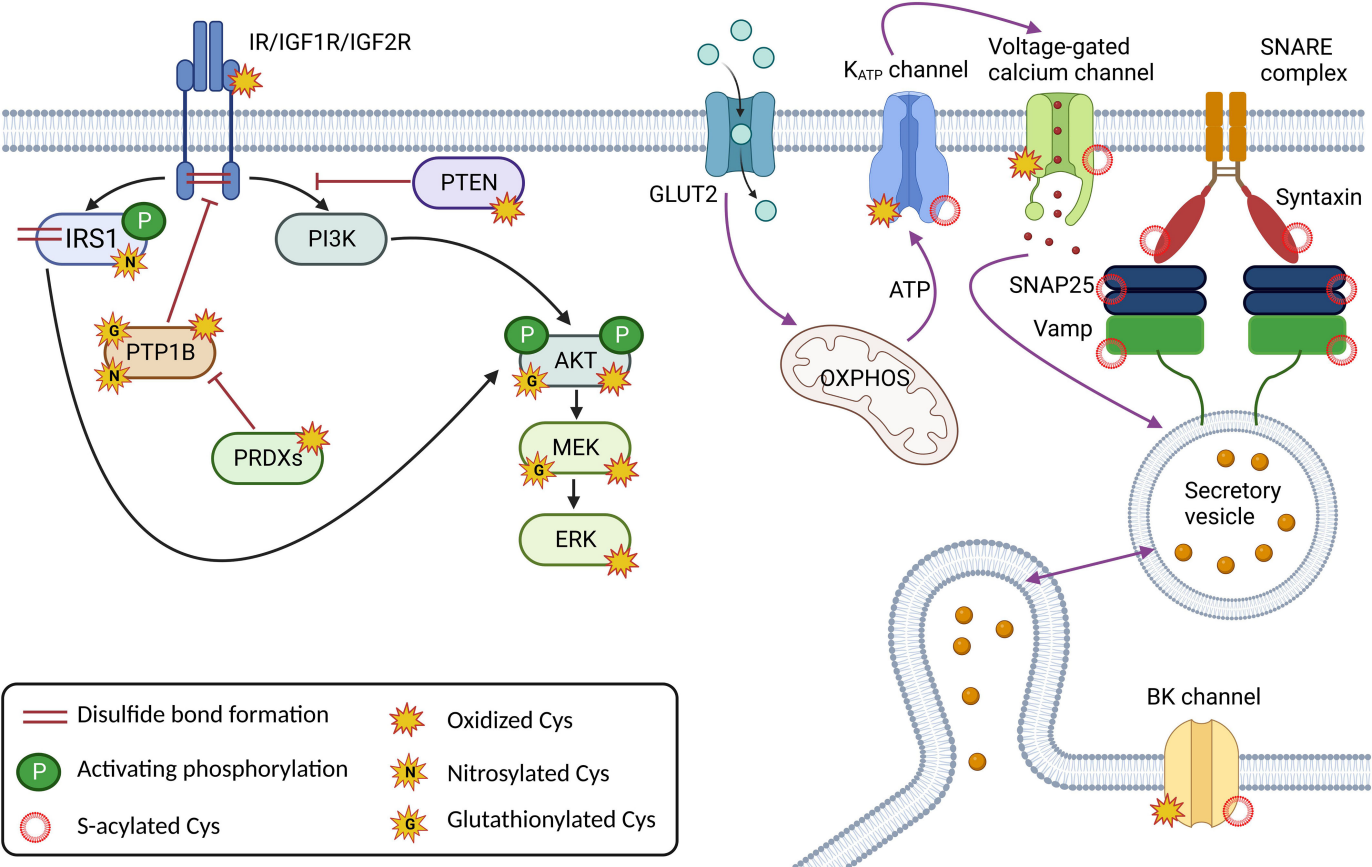
Major cysteine modifications in insulin and glucose signaling in β-cells; Correct redox signaling is an important requirement for healthy β-cells. Insulin receptor (IR) and Insulin-like growth factor receptors (IGF1/2R) create dimers via the disulfide bond formation, and oxidative modifications and nitrosylation are required for their activity. The redox signal is relayed by nitrosylation, glutathionylation, and/or oxidative modifications of downstream effectors such as the insulin receptor substrate (IRS1) and the kinases PI3K, Akt, MEK and ERK. The action of the kinases is regulated by the phosphatases PTP1B and PTEN, which are also subject to redox regulation. Components of the insulin secretory machinery, including ion channels at the plasma membrane such as K_ATP_, voltage-gated calcium channels and BK channels and proteins of the SNARE complex (SNAP25, Vamp, Syntaxin), are regulated by S-acylation. The Cys of ion channels are also oxidatively modified. This figure was created in Biorender.com (Toronto, Canada).

Insulin is an important regulator of energy metabolism, affecting proliferation and survival. Its receptors are present in many tissues important for glucose uptake and utilization, such as adipose tissue, skeletal muscle, and liver. However, they are also found in the brain, kidney, heart and pancreas. A functional insulin receptor (IR) ready for insulin binding requires the formation of disulfide bonds between the receptor´s subunits, and further signal propagation involves the formation of additional disulfide bonds that regulate its activity. These proteins are kinases and phosphatases as downstream signaling cascades trigger many intracellular phosphorylation events. Insulin receptor substrate-1 (IRS1) is a signaling protein that is phosphorylated by IR and activates downstream signaling pathways. Not only does it undergo cysteine oxidation to stabilize the molecule by forming cysteine bonds, but it is regulated by S-nitrosylation, which affects downstream insulin signaling through Akt phosphorylation. Several studies suggest an important role of IRS-1 S-nitrosylation in insulin resistance through the degradation of IRS1 via the ubiquitin-proteasome pathway (56–58). The effect of S-nitrosylation on IRS1 in adipocytes is more inhibitory for glucose uptake. S-nitrosylation decreases tyrosine phosphorylation of IRS1 and activation of Akt. This effect is thought to be mediated by inhibition of protein tyrosine phosphatases (PTPs) by S-nitrosylation, resulting in increased serine phosphorylation of IRS1 and decreased tyrosine phosphorylation (59, 60). Another example of redox regulation of insulin receptors has been shown for IGFs. IGF1 and IGF2 are growth factors that share significant structural homology with insulin and bind to IR. Apart from the role that cysteine residues in IGF1 and IGF2 play in their structural stability and activity, they may form disulfide bonds with other proteins, such as IGF binding proteins (IGFBPs), which may regulate the activity and availability of these growth factors in the extracellular space (61). In any case, IR phosphorylation is regulated by the well-characterized protein tyrosine phosphatase 1B (PTBP1B) (62). Direct oxidation of PTBP1B is rather slow (~10^1^ M^-1^. s^-1^) and it has been suggested that abundant peroxiredoxins having a higher affinity for oxidation, compete for H_2_O_2_ in cells (4, 63). The mechanism of PTBP1B oxidation occurs through the synergy of two independent mechanisms. First, bicarbonate/CO_2_ accelerates the oxidation of phosphatase and the same system facilitates the inhibition of peroxiredoxins by hyperoxidation, thus enabling the oxidation and inactivation of PTBP1B (63). Moreover, the activity of phosphatase is dependent on the pH in cells, and its activity increases at lower pH. This is consistent with the dependence of phosphatase oxidation on pH, which increases with higher pH (63, 64). Moreover, PTBP1B has been shown to be partially glutathionylated (at Cys215), resulting in decreased activity (65). Similarly, it has been suggested that S-nitrosylation reversibly inactivates PTBP1B, which may protect the protein from permanent inactivation by oxidative stress (66). Thus, the redox regulation of this PTBP1B is quite complex and largely depends on the metabolic state. S-glutathionylation has also been described for other downstream effectors of insulin signaling pathways, PI3K-Akt, Ras-MEKK1 (e.g. PTEN, IKKβ, NFκB, MEKK1, etc.) during the development of diabetes and some diabetic models (more in (67)), reducing their activity (**Figure 2**).

### 5.3 S-acylation in β-cells

S-acylation has been shown to be an important regulator of ion channels, vesicle trafficking, and small GTPAses, controlling many aspects of protein sorting, membrane localization, and lipid metabolism (68). S-acylated proteins of SNARE complex, including syntaxin, SNAP25, VAMP2 and synaptotagmin 1 are involved in the synaptic vesicle fusion machinery in neuronal cells (69) and also in pancreatic β-cells (70). K_ATP_ channel activity is strongly modulated by S-acylation, which causes channel opening, counteracting the ability of ATP to close the channel (71, 72). Stimulating glucose levels, however, are still able to close the channel, probably by rapid long-chain-CoA esterification to diacylglycerol (68, 73). S-acylation has also been shown to modulate the activity of voltage-gated Ca^2+^ channels (74) and BK channels (75), thereby affecting the electrical excitability of pancreatic β-cells by controlling action potential amplitude, depolarization, and repolarization that determine Ca^2+^ influx and insulin exocytosis (76) (**Figure 2**). Among other targets, adenine nucleotide translocase (ANT) has been shown to be inhibited by S-acylation, resulting in a decrease in the ATP : ADP ratio and increased ROS production (77).

## 6 Conclusion

It is obvious that not all Cys residues are the same and that the Cys residues that confer signaling properties to proteins are unique because of their position in the protein, the surrounding amino acids, protein cellular localization, and its redox environment. Although we have extensive knowledge of the location of cysteine modifications in individual proteins, we lack a functional understanding and interplay of the various cysteine modifications in a single protein and their consequences on the global scale. It is well known that β-cells require redox signaling through cysteine modifications for efficient glucose-stimulated insulin secretion and insulin signaling because of their sensitive redox homeostasis. However, metabolic overload and imbalanced redox homeostasis may lead to the development of T2D. This increases the need for research in this area as a potential intervention in the treatment of diabetes development.

## Author contributions

Conceptualization: BH and LP-H; Writing the original manuscript: BH and LP-H; Manuscript review and editing: BH and LP-H; Funding acquisition: LP-H. LP-H is the guarantor of this work. All authors contributed to the article and approved the submitted version.
