# Corrigendum: Cysteine residues in signal transduction and its relevance in pancreatic beta cells

**DOI:** 10.3389/fendo.2024.1399741

**Published:** 2024-03-20

**Authors:** Blanka Holendova, Lydie Plecita-Hlavata

**Affiliations:** Laboratory of Pancreatic Islet Research, Institute of Physiology, Czech Academy of Sciences, Prague, Czechia

**Keywords:** cysteine, thiol, pancreatic beta cells, posttranslational modifications, redox signaling

In the published article, there was an error in the Funding statement. The European Union Next Generation EU was not mentioned in the statement. The correct Funding statement appears below.

This work was supported by the Grant Agency of the Czech Republic, grant No. 22-11439S to LP-H and by the project National Institute for Research of Metabolic and Cardiovascular Diseases (Programme EXCELES, ID Project No. LX22NPO5104) - funded by the European Union Next Generation EU.

The authors apologize for this error and state that this does not change the scientific conclusions of the article in any way. The original article has been updated.

